# Prenatal diagnosis of a terminal chromosome 1 (q42-q44) deletion: original case report and review of the literature

**Published:** 2016-06-27

**Authors:** C Van Linthout, V Emonard, JS Gatot, X Capelle, F Kridelka, P Emonts, MC Segghaye

**Affiliations:** Department of Obstetrics and Gynaecology, University of Liège, 4000 Liège, Belgium.; Department of Clinical and Human Genetics, University of Liège, 4000 Liège, Belgium.; Department of Paediatrics, University of Liège, 4000 Liège, Belgium.

**Keywords:** Chromosome 1 deletion, facial dysmorphy, microcephaly, non compaction of myocardium, growth retardation

## Abstract

Terminal chromosome 1q deletion is rarely reported but causes typical malformations that have been well described in childhood.

Clinical features include facial dysmorphy, growth and/or psychomotor retardation, brain agenesis or hypoplasia of the corpus callosum, epilepsy and occasional urogenital or cardiac malformations.

The diagnosis of this condition is usually made at birth. The rare cases of antenatal diagnosis were based on microcephaly and growth retardation. In the present case, the foetus presented with an hypoplasia of the corpus callosum, a dysmorphic profile and a single umbilical artery. The foetal echocardiography suggested a non- compaction of the left ventricular myocardium. No microcephaly or growth retardation were noted. We compare our antenatal findings to those described in the literature with the aim to better define the antenatal phenotype of the terminal chromosome 1 deletion syndrome.

## Case report

**Fig. 1:**
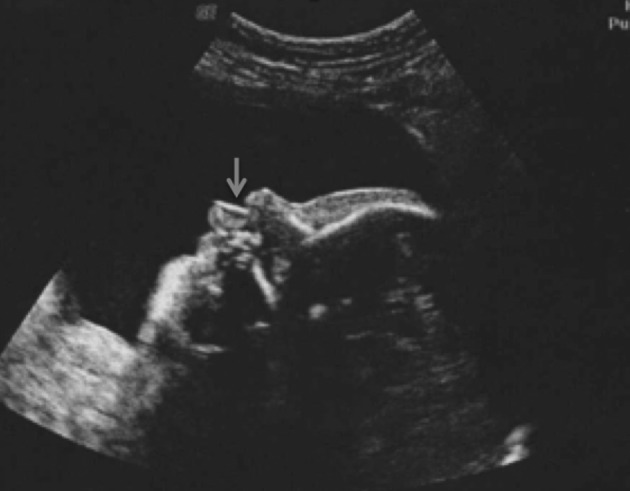
— Dysmorphic profile with long upperlip

**Fig. 2:**
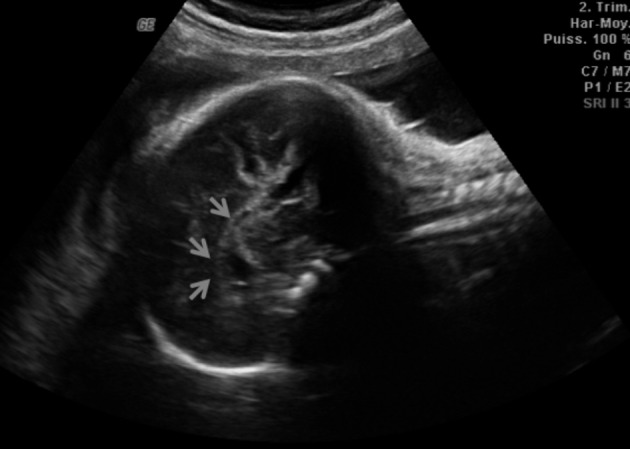
— Hypoplasia of corpus callosum

**Fig. 3:**
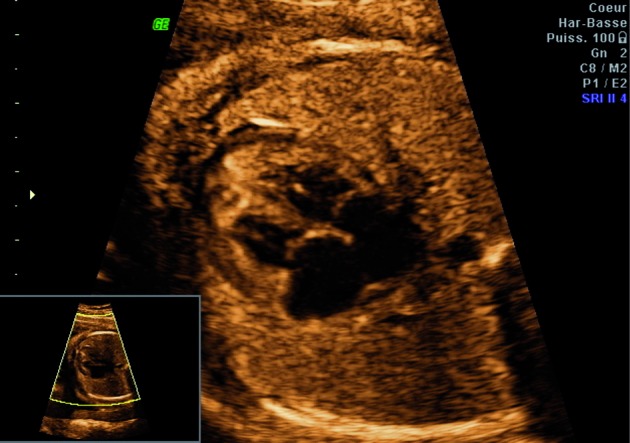
— Abnormal thickened myocardium

We report the case of a 17-year-old primigravida with a normal medical history who first presented to the obstetrical outpatient clinic at 16 weeks of gestation. As a consequence, no nuchal translucency could be measured on ultrasound at the appropriate 12-week term. The second trimester biochemical triple screen test showed a low risk for trisomy 18 and 21. A routine 23 weeks ultrasound examination demonstrated a female foetus with a growth curve at the 25th percentile. The profile was dysmorphic with a marked retrognatism and a long upper lip with a smooth philtrum (Fig. 1). The nasal bone was normal and the corpus callosum hypoplasic (Fig. 2). The heart was «bulging» with a thickened myocardium (Fig. 3). We observed a single umbilical artery together with a pathological left uterine artery doppler. The volume of amniotic fluid was normal.

**Fig. 4:**
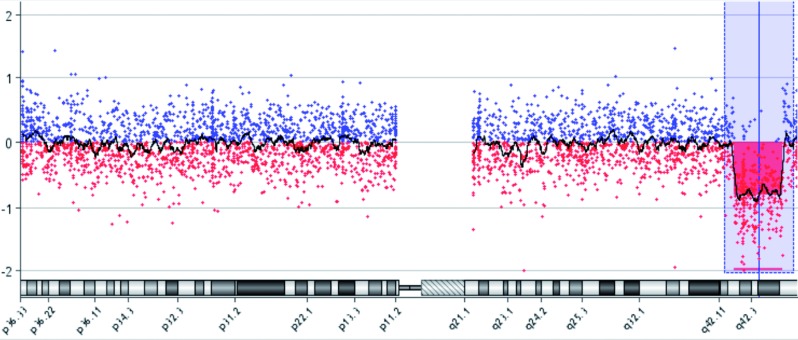
— Terminal deletion of the long arm of chromosome 1

An amniocentesis was performed and showed a female karyotype with a terminal deletion of the long arm of chromosome 1 (15.58 Mb deletion in region 1q42.13-q44) (Fig. 4). Parental karyotypes were verified to exclude an inherited translocation but the karyotypes of both parents turned out to be normal. The couple was referred for genetic counselling but, despite the very poor foetal prognosis, the couple decided not to allow an interruption of the pregnancy.

Future ultrasound findings at 32.4 weeks of gestation demonstrated a polyhydramnios. The cardiac function was normal and the growth curve remained on the 25th percentile. No microcephaly was noted with a cranial perimeter measured at 288.5 mm (48th percentile using the CFEF curves).

At 34 weeks, a premature rupture of membranes complicated by a chorioamniotitis led to a caesarean section. A baby girl was delivered with a birth weight of 1990 g (Percentile 40). The foetus was born in a state of apparent death, subsequently resuscitated and intubated. Facial dysmorphy was present with micrognathia, low set ears, a short neck, hypertelorism and a smooth and long philtrum. The corpus callosum was hypoplastic and the cardiac echography confirmed the abnormally thickened myocardium without structural abnormalities. The baby died 7 days after birth due to cardiorespiratory failure. The parents refused an autopsy.

## Review of the literature

To date, 30 children with microscopically visible terminal deletion of chromosome 1q have been reported in the literature (De Vries et al., 2001). The clinical syndrome associated with this karyotypic condition includes moderate to severe growth and psychomotor retardation, epilepsy, microcephaly and central nervous system anomalies (hypoplasia or agenesis of the corpus callosum, ventriculomegaly, Arnold-Chiari type 1 malformation) (Wagner et al., 2011). Patients show typical facial features (micrognathia, short neck, low set ears, long upper lip). Other malformations include urogenital (hypospadia) and cardiac anomalies (tetralogy of Fallot, atrial and ventricular septal defect) (Nagamani et al., 2012).

Very few cases of prenatal diagnosis have been reported and the antenal echographic findings that should suggest a terminal deletion of chromosome 1 have not been standardized yet. A summary of the reported antenatal features is shown in Table I. Most diagnoses are made in the second and third trimester of gestation. Only Wagner et al. (2011) described a case diagnosed at the occasion of the first trimester ultrasound.

Antenatal growth retardation and microcephaly seem to be present in all cases described in the literature but are not specific for a 1 q deletion; however craniofacial features (microgenia, nasal bone hypoplasia or absence of the nasal bone), brain malformations (ventriculomegaly, hypoplasia or agenesis of corpus callosum) and cardiac anomalies (septal defect, pulmonary atresia with intact septum) can suggest this genetic abnormality, especially if they are associated. The hypospadia and the cleft palate were not reported prenatally. Hydramnios and a single umbilical artery was described before in one single case. In this case report, growth retardation and microcephaly were not present and have not led to the prenatal suspicion of 1q deletion. Although microcephaly has been described to be progressive (Hill et al., 2007), it was not present at 32 weeks.

Based on the analysis of 15 cases with 1 q terminal deletion, Johnson et al. (1985) reported the characteristic facial appearance and pattern of associated malformations in children with 1 q 42-43 deletion (Johnson et al., 1985). Growth retardation is found at birth only in 4 cases but all children developed growth retardation after birth. In all cases at birth microcephaly was present.

In our patient, the analysis of the foetal profile has allowed us to highlight a long upper lip with a smooth philtrum and retrognathia. Only De Vries et al. (2001) and Wagner et al. (2011) describe an abnormal profile associated with this condition.

The cardiac anomaly seems to be consistent with a non-compaction of the left ventricular myocardium (LVNC). Kanemoto et al. (2006) reported a newborn infant with LVNC associated with interstitial 1q43-q43 deletion. The deleted region included the locus for the RyR2 gene (cardiac ryanodine receptor type 2 gene). It was the first case reporting an association between LVNC and terminal 1q deletion.

Postanatal echocardiography confirmed prominent trabeculations with deep intertrabecular recesses in the left ventricle without septal defect.

In conclusion, only a few cases of terminal 1q deletion syndrome have been diagnosed antenatally and the malformations detected do not appear to define a specific 1q deletion foetal phenotype.

Growth retardation and microcephaly may not develop during foetal life and are not always present in the context of terminal 1q deletion. The facial dysmorphism is, oppositely, systematically described in affected children.

We therefore recommend to perform a detailed analysis of the foetal profile at routine ultrasound to search anomalies such as microgenia, retrognathia, philtrum anomaly, hypoplasia or absent nasal bone. We consider that non-compaction of the left ventricular myocardium can be associated with terminal chromosome 1q deletion.
